# Optically Determined Hole Effective Mass in Tin-Iodide Perovskite Films

**DOI:** 10.1021/acsenergylett.5c02283

**Published:** 2025-08-28

**Authors:** Vincent J.-Y. Lim, Marcello Righetto, Michael D. Farrar, Thomas Siday, Henry J. Snaith, Michael B. Johnston, Laura M. Herz

**Affiliations:** † Department of Physics, Clarendon Laboratory, 6396 University of Oxford, Parks Road, Oxford OX1 3PU, U.K.; ‡ School of Physics and Astronomy, 1724 University of Birmingham, Birmingham B15 2TT, U.K.

## Abstract

Tin-halide perovskites currently offer the best photovoltaic performance of lead-free metal-halide semiconductors. However, their transport properties are mostly dominated by holes, owing to ubiquitous self-doping. Here we demonstrate a noncontact, optical spectroscopic method to determine the effective mass of the dominant hole species in FASnI_3_, by investigating a series of thin films with hole densities finely tuned through either SnF_2_ additive concentration or controlled exposure to air. We accurately determine the plasma frequency from mid-infrared reflectance spectra by modeling changes in the vibrational response of the FA cation as the plasma edge shifts through the molecular resonance. Our approach yields a hole effective mass of 0.28*m*_*e*_ for FASnI_3_ and demonstrates parabolicity within ∼100 meV of the valence band edge. An absence of Fano contributions further highlights insignificant coupling between the hole plasma and FA cation. Overall, this approach enables noncontact screening of thin-film materials for optimized charge-carrier transport properties.

## Graphical abstract

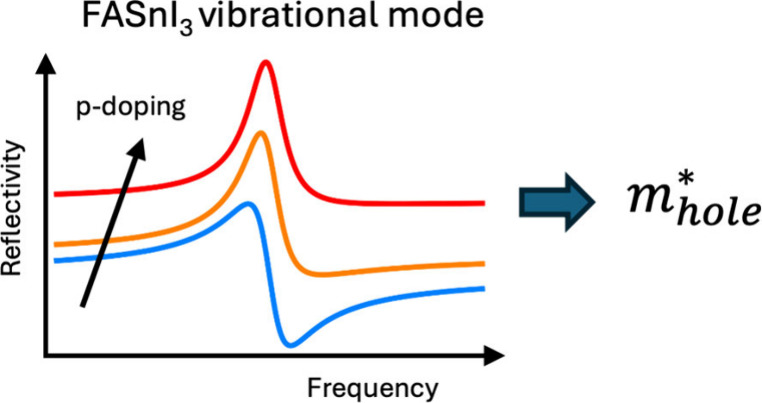

Lead-free halide perovskites have emerged as an environmentally compatible alternative to lead-halide perovskites, with potential applications ranging from photovoltaics, photocatalysis, to field-effect transistors.^1–5^ In this family of materials with stoichiometry ABX_3_, toxic Pb­(II) B cations are replaced by isoelectronic metal cations, such as Sn­(II) or Ge­(II), or, in double perovskites of stoichiometry A_2_BB’X_6_, by heterovalent cation pairs, such as Ag­(I)–Bi­(III) or Ag­(I)–Sb­(III).^6,7^ Beyond addressing toxicity constraints, the vast chemical space available for lead-free halide perovskites has enabled extending the bandgap engineering range for multijunction solar cell applications^8–10^ and developing further emerging properties of these metal-halide semiconductors, such as photo­(electro)­catalytic^3^ and white-light emission properties.^11^ However, replacing Pb­(II) cations while preserving the excellent optoelectronic properties of lead-halide perovskites^12^ has proven an extraordinary challenge.^13^ Limited charge-carrier transport, originating from both intrinsic (i.e., band dispersion and phonon coupling) and extrinsic (i.e., traps and grain boundaries) effects, is currently a major challenge for lead-free perovskites. Therefore, understanding the role of metal cation substitution in transport properties is critical for developing efficient lead-free perovskite materials.

Tin-halide perovskites of stoichiometry ASnX_3_ have to-date proven to be the best-performing lead-free perovskite semiconductors by some margin, with photovoltaic conversion efficiencies in excess of 15% now having been reported.^14^ Here, the halide X-site is typically chosen to be iodide (I^–^) for lowest band gaps, and the A-site may be occupied by formamidinium (FA^+^), methylammonium (MA^+^), cesium (Cs^+^), or a mixture thereof,^13,15–18^ with FA being increasingly popular owing to its superior thermal stability compared with MA.^19^ Early studies on pressed powders of tin iodide perovskites reported promising Hall mobilities of up to a few hundred cm^2^/(V s) but noted that these materials displayed strong p-type conduction.^15–18^ Further advances in materials processing yielded thin films of high optoelectronic quality,^5,20–22^ triggering implementation in devices such as solar cells^14^ and light emitters, however, issues with undesirably large background hole densities remained.^23,24^ Such effects ultimately arise from tin iodide perovskites exhibiting lower spin–orbit coupling than their lead-based counterparts (tin being lighter than lead),^22,25^ and therefore reduced ionization energies.^26^ Tin vacancies are thus easily formed,^17,26,27^ which in turn creates a locally iodine-rich environment that promotes the oxidation of Sn^2+^ to Sn^4+^ and chemical conversions e.g. to SnO_2_ or SnI_4_.^26,27^ Such links between tin vacancy formation, tin oxidation and chemical conversion unfortunately also open routes to material decomposition in air and water.^19,28^ From an electronic perspective, tin vacancies generate defect levels close to the valence band edge^26^ which effectively capture and extract electrons from the valence band, generating high hole densities^15,20,29^ often in excess of 10^18^ cm^–3^. Such excess holes may rapidly recombine with any photogenerated electrons and limit mobilities through the introduction of additional scattering processes. Significant advances in tin-halide perovskite processing have been made to minimize such unintentional self-doping, based e.g. on the use of additives to act as reducing agents or tin sources, control of crystallization, or partial ion substitution.^30,31^ The most popular strategy has been the use of SnF_2_ additive^13,29,32^ to create a Sn-rich environment that suppresses tin vacancy formation. However, despite such efforts, the optoelectronic properties of tin-halide perovskites ultimately still remain dominated by a single charge-carrier species: holes in the valence band.

While charge-carrier recombination and transport has been much examined in thin films of tin-halide perovskites,^20,32,33^ more fundamental material parameters, such as the values of the individual electron and hole effective masses, remain under debate. Experimentally, individual electron or hole masses can be hard to determine from all-optical measurements best suited to the investigation of thin films. Magneto-optical studies yielding values for reduced electron–hole masses have been widely applied to metal halide perovskites,^34^ including some mixed lead–tin iodides,^35^ but measurements for tin-only perovskites have been challenging. Optical measurements on tin-only halide perovskite films have generally been hampered by the effects of self-doping which causes very broad absorption spectra, as well as film inhomogeneity that causes strong light scattering, and material instability reducing data acquisition times.^35^ From a theoretical perspective, the charge-carrier effective masses are inversely proportional to the calculated curvature of the electronic bands, with relativistic effects playing a role.^13^ For tin-iodide perovskites, the homovalent replacement of Pb­(II) with Sn­(II) cations—which have an analogous electronic *n*s^2^ configuration—preserves the high electronic dimensionality (i.e., the connectivity of the orbitals comprising the band edges) of the material. Here, the Sn–I bond lies at the heart of the tin iodide perovskite: on the one hand, the conduction band comprises antibonding Sn 5p – I 5p orbitals, while on the other hand, the valence band is comprised of the hybridized antibonding orbitals from Sn 5s and I 5p states.^13,36^ First-principles calculations from Umari et al. have shown that the shallower and more active Sn 5s^2^ lone-pair states (with respect to the Pb 6s^2^ lone-pair states) yield more dispersive valence bands.^22^ However, theoretical computational studies have reported a surprisingly wide range of values for the effective masses of electrons and holes. While some studies report moderate values between 0.1 and 0.3*m*_*e*_, where *m*_*e*_ is the electron rest mass,^22,37–39^ others extend from as low as 0.05 to as high as 1.03*m*_*e*_.^36,40,41^ Therefore, despite the dominance of holes in tin halide perovskite films, an accurate determination of their masses remains elusive.

In this Letter, we provide an accurate determination of the hole effective mass for the archetypal tin-halide perovskite, FASnI_3_, by deploying a noncontact optical spectroscopic method to a series of FASnI_3_ thin films with different background hole densities. Specifically, we deduce the hole mass from a novel approach that extracts the plasma frequency accurately from mid-infrared reflectance spectra, by modeling changes in the vibrational response of the FA cation as the plasma edge shifts through its line shape with increased background hole density. To ensure high accuracy of mass determination, we utilize changes in SnF_2_ additive concentration during film fabrication as well as subsequent timed air exposure in order to vary the hole density across a wide range of values, monitored by noncontact THz conductivity measurements. Our approach yields a hole effective mass of 0.28*m*_*e*_ for FASnI_3_ and further establishes a near-parabolic valence band up to energies of at least 100 meV above the band edge. This approach demonstrates the power of an optical approach toward determining individual charge-carrier masses in thin films for photovoltaic applications, which will aid the rapid design and exploration of materials with optimized charge-carrier transport.

We investigated a series of FASnI_3_ thin films deposited onto z-cut quartz substrates, with the initial background density of holes being controlled by tuning the concentration of the SnF_2_ additive in the precursor solution before spin-coating onto z-cut quartz (Supporting Information (SI) Section 1). We determined the hole density across the thin-film series by measuring the thin-film conductivity spectra of the films by means of terahertz-time domain spectroscopy (THz-TDS), displayed in Figure 1a and SI Figures S2 and S3. We assumed a comparatively negligible dopant density for FASnI_3_ thin films with 20% SnF_2_ additive, for which the measured THz spectrum is predominantly associated with the broad absorption peaks of tin-halide optical phonon modes, as reported earlier.^32^ As the SnF_2_ concentration was reduced from 20% to 0%, we observed a significant increase in the film conductivity *σ*_*dark*_ (Figure 1a), as a result of the additional free hole density arising from self-doping. We find increasing SnF_2_ concentration is associated with general improvements in optoelectronic film quality (see e.g. Figure S4 in SI) in agreement with other studies highlighting beneficial effects of SnF_2_ addition up to 20%.^42–44^ As shown previously,^28,32^ we are able to convert such dark conductivity values to background hole densities in combination with measurements of the THz mobility determined through the optical-pump terahertz-probe (OPTP) technique (Figure S4)—see SI Section 2.3 for further details. The hole mobility *μ*_*h*_ was determined from the THz mobility value obtained from OPTP (Figure S4) based on comparable electron and hole mobilities^22,45^ and the hole density *p* determined via the equation *σ*_*dark*_ = *peμ*_*h*_. The resulting hole density values for the thin-film series (Figure 1b) show a monotonic increase in doping density with decreasing SnF_2_, and fall into a density regime where holes exist as free charge carriers.^46^ In addition, we were able to achieve finer steps in hole density by controlled exposure of the films to ambient air for discrete amounts of time, which further increase the p-doping in the material, as can be seen in Figure 1c.^28^

**Figure 1:**
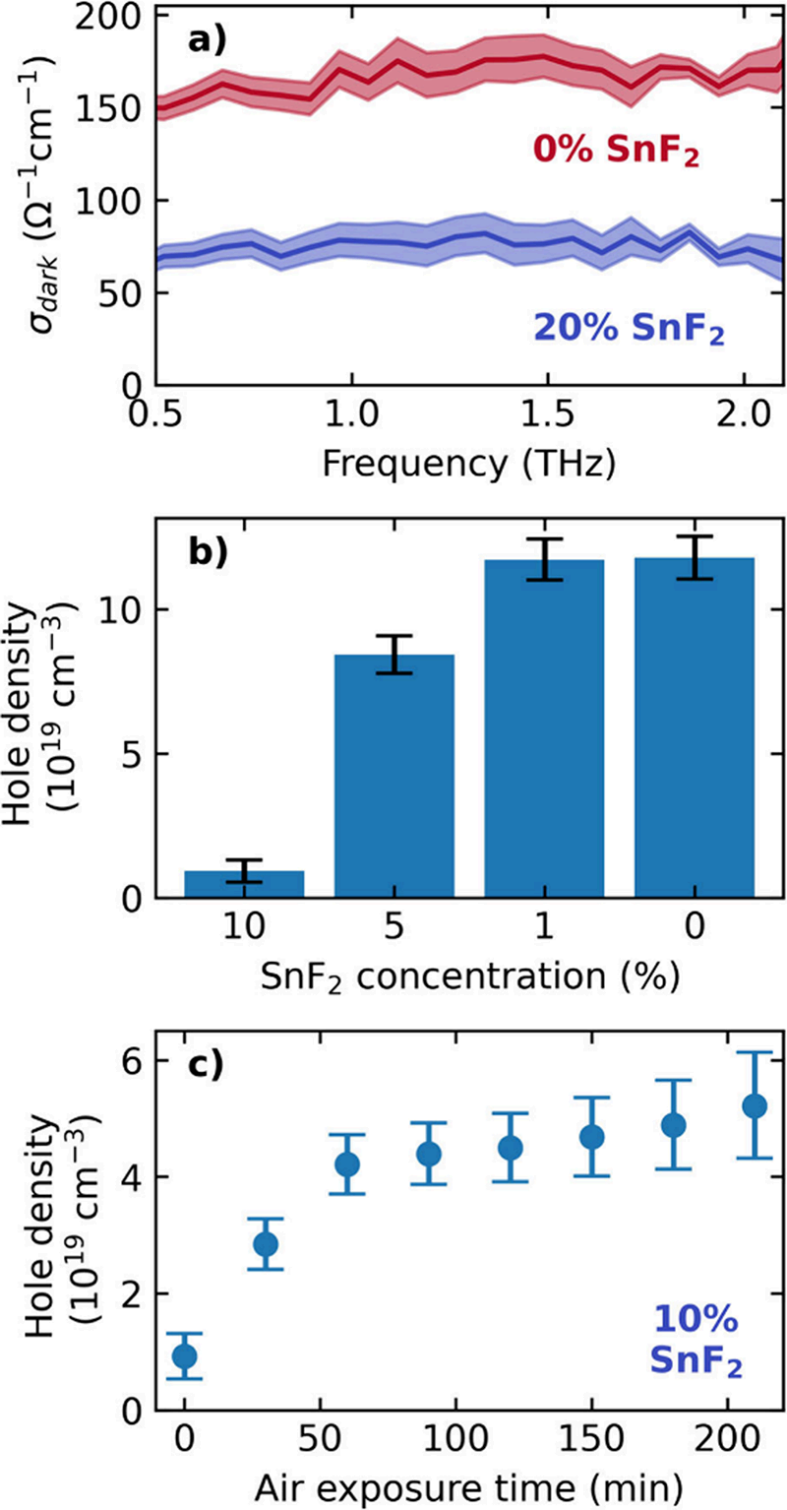
(a) THz dark conductivity spectra of FASnI_3_ films processed with 0% and 20% SnF_2_ additive. (b) Derived hole density in FASnI_3_ films with 10%, 5%, 1% and 0% SnF_2_ additive. (c) Hole density in FASnI_3_ film (with 10% SnF_2_ additive) as a function of increasing time in ambient air (temperature: 19 °C; ambient humidity: 45%).

For a bulk semiconductor, a conventional and straightforward method of measuring the effective mass of a single charge carrier can be the measurement of the plasma frequency of the doped semiconductor, following determination of the doping density from Hall effect measurements.^15,47^ However, for thin films, application of this method is practically hampered, in particular for tin-halide perovskites for which thin-films absorption features are particularly broad, thus hindering the clean observation of a plasma edge.^35^ We posit here that by investigating changes in the line shape of IR-frequency vibrations of the organic FA cation as the plasma frequency sweeps through the vibrational peaks, we can accurately determine the plasma frequency. Here, we model the dielectric function of a doped semiconductor hosting a vibrational mode as a sum of a plasma response and a Lorentz oscillator term:^48,49^

1 $$\epsilon =\epsilon_{\inf}\left(1-\frac{\omega_{p}^{2}}{\omega^{2}+i\omega \Gamma }\right)+\frac{A^{2}}{\omega_{FA}^{2}-\omega^{2}-i\omega \Gamma_{FA}}$$

where ω is the angular frequency (with ω = 2*πν*, and *v* being the frequency of the incident light indicated on the *x*-axes of Figure 2a,b), *ϵ*_*inf*_ is the dielectric constant at infinite angular frequency (with respect to the observation window), *ω*_*p*_ is the plasma frequency, Γ and Γ_*FA*_ are the broadening factors for the charge-carriers plasma and the FA vibrational mode, respectively, *A* is the oscillator strength of the vibration, and *ω*_*FA*_ is the angular frequency of the FA internal vibrational mode. The plasma frequency is defined by ω_*p*_^2^ = *pe* ^2^/ϵ_0_ *ϵ*_*inf*_ *m*_*eff*_, where is the doping density, ϵ_0_ is the vacuum permittivity, and *m*_*eff*_ is the effective mass of the dopant charge carrier, i.e., the hole in this instance. Within this model, the reflectance line shape of the vibrational mode associated with a doped semiconductor is expected to change significantly, as demonstrated in Figure S6 which plots example solutions for eq 1. Such line shape change originates from the summation of the plasma mode and the Lorentz oscillator terms, with the phase of the overall dielectric constant markedly changing as the plasma frequency shifts through the frequency of the FA vibrational mode.

**Figure 2:**
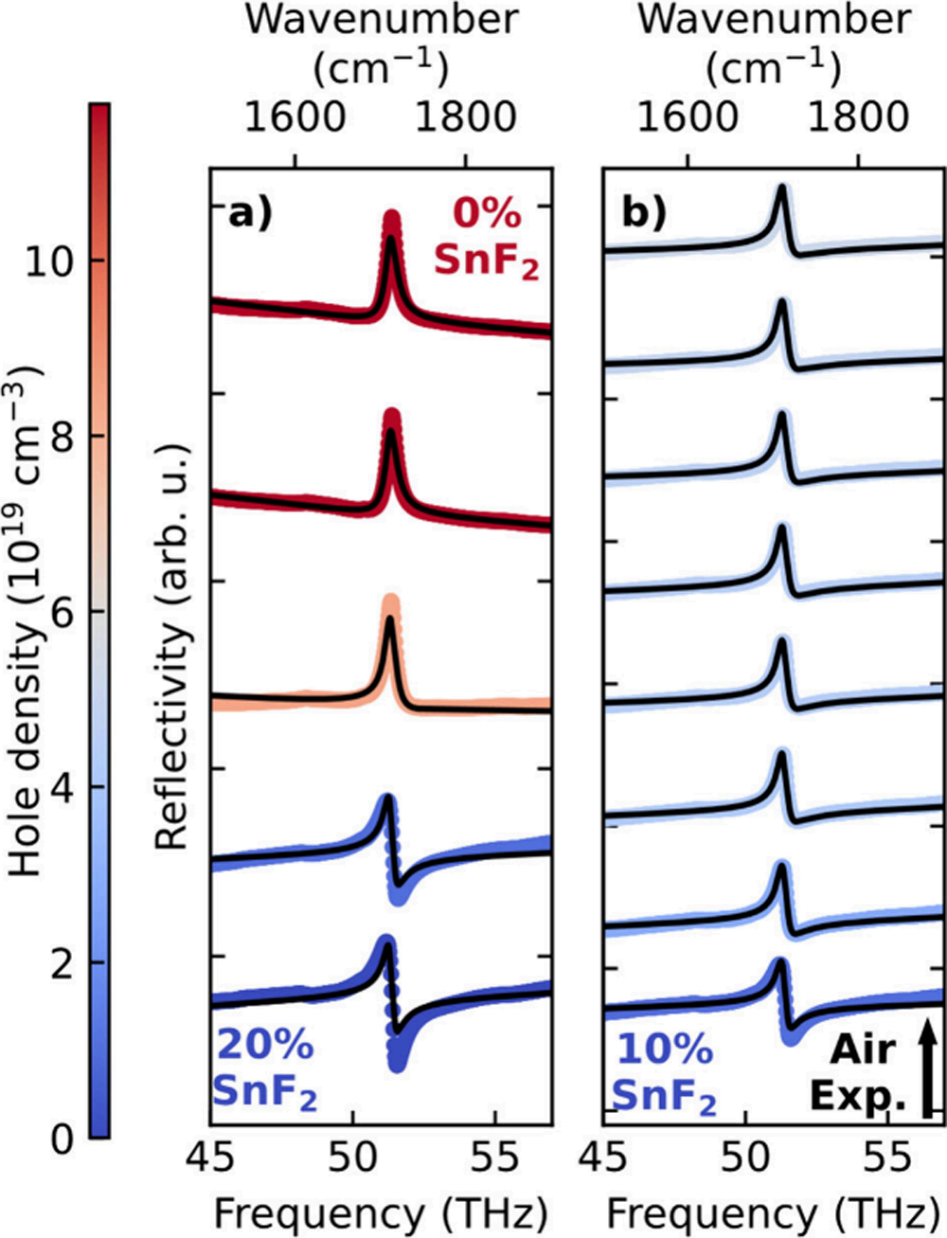
Reflectance spectra as a function of IR frequency *v* for FASnI_3_ films with (a) different amounts of SnF_2_ additive and (b) 10% SnF_2_ additive and air exposure time increasing from 0 to 210 min, in steps of 30 min. As previously shown,^28^ air-exposure over the first 300 min has the predominant effect of increasing hole doping, with secondary-phase formation only commencing at longer times. Plots focus on the CN stretching mode of the FA cation. Black lines correspond to fits based on eq 1. Note that the *x*-axis reflects standard frequency *v* while eq 1 and the plasma frequency are given in terms of angular frequency *ω* = 2*πν*, as conventional.

We find that for FASnI_3_ films, such line shape changes can indeed be clearly observed for the CN stretching mode of the FA cation at ∼ 1710 cm^–1^ as the hole density is varied (Figure 2a,b). We measured the spectral line shape in reflectance,^50^ using the Fourier Transform Infrared (FTIR) technique, as detailed in SI Section 2.1. The line shape of the FA mode gradually but significantly changes as the hole density in the FASnI_3_ films increases, either through decreases in SnF_2_ concentrations (20, 10, 5, 1 and 0%, as shown in Figure 2a) or when FASnI_3_ films are exposed to air (Figure 2b for a FASnI_3_ film with 10% SnF_2_ addition) which induces finely tuned changes in hole density. We have fitted these reflectance spectra based on the dielectric function captured by eq 1 with the resulting fits shown as black lines in Figure 2a,b. Here, for increased accuracy, the parameters *ϵ*_*inf*_, Γ, *A*, ω_0_, and Γ_*ph*_ were only allowed to vary globally and solely *ω*_*p*_ and a linear background term were permitted to vary between different spectra (see SI Section 3 for further details and discussion of the linear background term arising from minor variations between reflection from the substrate and surface scattering from different films). The resulting values extracted for the global parameters are shown in Table S1 in the SI. We briefly comment on the extracted value of *ϵ*_*inf*_ = 8.0 ± 0.9, which is in good agreement with literature reports ranging between 6.6 and 8.85.^37,40,41^ We note that the term *ϵ*_*inf*_ can be somewhat ambiguous depending on the high-frequency limit of which resonance it refers to, with common usage referring to the near-IR range immediately below the electronic bandgap.^51^ Our study focuses on molecular vibrational modes of the FA cation in the mid-IR range. Such vibrational modes in the mid- to near-IR range have been shown to only contribute marginally to the overall dielectric function,^52,53^ and hence our value of *ϵ*_*inf*_ is similar to but slightly at the higher side of commonly reported values.

We further note that, intriguingly, the line shapes we report here resemble those for a Fano resonance,^54^ a phenomenon arising from interference between a background of continuum states and a resonant (discrete) scattering process. This phenomenon is caused by weak coupling between the continuum and discrete states, and is revealed as a characteristic asymmetric line shape in either absorption or reflection spectra.^54,55^ The Fano resonance line shape depends on the ratio of scattering between the two states, characterized by the Fano parameter *q*.^56^ Assigning the observed reflectance response and their evolution with hole density to a Fano resonance with varying Fano parameter *q* would imply that a direct coupling exists between the hole plasma (the continuum) and the CN stretch mode (the discrete state).^54^ Interestingly, there have been some reports of coupling between charge carriers and internal A-cation modes, possibly mediated by lead-halide cage modes,^57,58^ and therefore such Fano resonance cannot be fully ruled out. However, our model captured by eq 1, which simply sums the plasma and oscillator responses to describe the dielectric function, will hold regardless of the presence of a Fano resonance. While a Fano-type resonance could, in principle, contribute as a further term to this approach, we note that our model already describes the experimental reflectance spectra very well, even without such an addition. We therefore conclude that any direct coupling between holes and internal A-cation modes can be only of relatively minor magnitude.

The square of the plasma frequency extracted from these fits shows a linear dependence on the hole density *p* (see Figure 3), as expected from the relation ω_*p*_^2^ = *pe* ^2^/*ϵ*_0_ *ϵ*_*inf*_ *m*_*eff*_.^59^ The clear passage through the origin further supports our assumption of comparatively negligible hole densities encountered in the FASnI_3_ thin film with 20% SnF_2_ addition. Moreover, the linear behavior demonstrates that the parabolic approximation holds (i.e., constant mass) for the valence band of FASnI_3_ even at doping densities as high as 10^20^ cm^–3^ for which the valence band will be significantly depleted (within the order of 100 meV at these doping densities^20^). We are thus able to extract the hole effective mass of *m*_h_^*^ = 0.28 ± 0.05*m*_*e*_ from linear fits to these data with good accuracy. We note that this value is at the higher end of the range of typical predictions reported for computational calculations of the hole mass in ASnX_3_ perovskites (*m*_h_^*^ = 0.1–0.3*m*_*e*_),^22,36–41^ which underlines the need for experimental findings. We further note that our values are comparable to the effective hole mass of around *m*_h_^*^ = 0.2–0.3*m*_*e*_ deduced from bandstructure calculations and angle-resolved photoelectron spectroscopy for lead-halide perovskites.^22,60–62^

**Figure 3:**
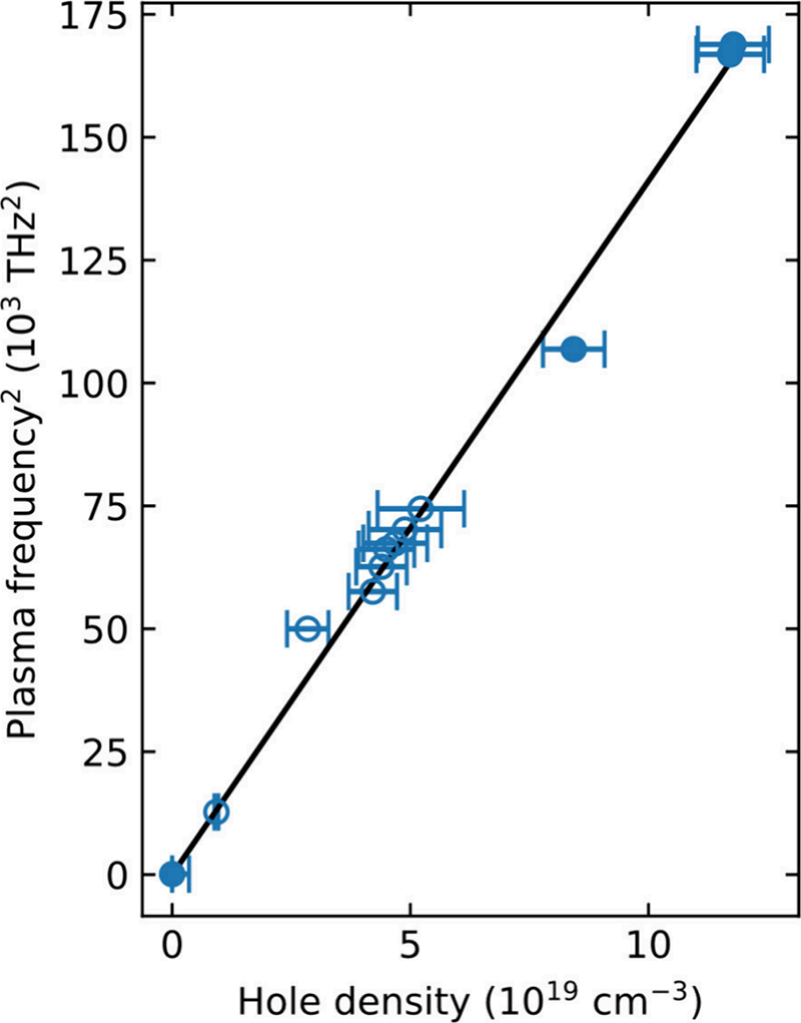
Square of the (angular) plasma frequency plotted as a function of the hole density, obtained from fits shown in Figure 2a,b, which demonstrates a linear dependence indicative of parabolic bands. Hollow circles correspond to data derived from the FASnI_3_ film with 10% SnF_2_ additive measured after different amount of air exposure (as shown in Figure 2b), and solid circles correspond to FASnI_3_ films with different amounts of SnF_2_ addition (as shown in Figure 2a).

Our experimentally determined hole effective mass value for FASnI_3_ has clear implications on the current understanding of charge-carrier transport in tin-iodide perovskites. A lower effective mass of charge carriers in tin-halide perovskites, obtained from first-principles simulations, has generally been quoted as the reason for higher mobilities in tin-halide perovskites compared to lead-halide perovskites.^29,45^ However, our results suggest that other factors, such as coupling of charge carriers to phonons, may be responsible for the higher mobility. Within the Fröhlich model, the charge-carrier mobility is inversely proportional to the coupling coefficient α = ϵ_*Fr*_^–1^(*Ry*/*ℏω*_*LO*_)^1/2^(m*/*m*_*e*_)^1/2^, where *ω*_*LO*_ is the LO phonon frequency, *Ry* = 13.606 eV the Rydberg constant, and ϵ_*Fr*_^‑1^ = ϵ_*inf*_^–1^ – ϵ_*static*_^–1^.^29,51^ Factors other than the charge-carrier masses may therefore affect mobilities; for example, the lighter B-cation in tin-halide perovskites (compared to their lead-based counterparts) may lead to higher values of *ω*_*LO*_ and lower Fröhlich parameter α. In addition, any changes in the polar nature of the tin-halide bond may also be a factor. We note that some dependence of electron–phonon coupling on doping density has been reported,^46^ however our linear dependence of plasma frequency on doping density suggests that we are in the Drude regime associated with a free-charge response. We further note that some of the earlier reports^18^ of tin iodide perovskites exhibiting room temperature mobilities of several hundred or thousand cm^2^/(Vs) in single-crystal specimens have not been repeatable for thin films.^20^ Such differences may partly arise from inaccuracies arising when combining Hall coefficient and resistivity measurements across different specimens, and partly be related to heavy doping leading to a reduction in charge-carrier mobility as a result of carrier–carrier scattering and interactions with ionised impurities (e.g., tin vacancies).^20,29,63^ As such, charge-carrier mobility values reported for thin films of tin- and lead-iodide perovskites are therefore often ultimately found to be within the same order of magnitude,^20,28^ i.e. a few tens of cm^2^/(V s).

In conclusion, our study reports the hole effective mass of FASnI_3_ thin films determined experimentally through noncontact optical spectroscopy. Our work utilizes a FASnI_3_ thin film series with finely controllable hole density, which is tuned by either changes in the SnF_2_ additive concentration during film fabrication or by exposing the film to the air to induce further tin vacancy formation and oxidation. We quantify the hole density through an all-optical approach by combining optical conductivity measurements from THz-TDS and mobility measurements from OPTP. We further determined the plasma frequency accurately by monitoring the IR reflectivity spectra across the frequency range of an internal vibrational mode of the FA cation. As the plasma frequency is swept past the resonance of the sharp FA mode with increasing hole density, clear changes to the line shape are observed and modeled based on a combined dielectric function capturing both the plasma and the molecular response. From the extracted plasma frequencies, we were able to determine a hole effective mass of 0.28 ± 0.05*m*_*e*_ for FASnI_3_. This value appears similar to that reported for lead-halide perovskites, suggesting that any potential differences in mobilities may ultimately arise from changes in electron–phonon coupling. In addition, we note that our simple model highlights the absence of a Fano resonance in these spectra, i.e. any direct coupling between the continuum of hole states and the sharp A-cation molecular resonance appears to be insignificant. Overall, our results provide novel fundamental insight into the electronic properties of tin-halide perovskites, which currently offer the most promising photovoltaic performance within the emerging group of lead-free metal halide semiconductors.

## Supplementary Material



## Notes

Sample fabrication details, experimental details of spectroscopic techniques and further characterization data, including mid-IR reflectance and optical absorption, terahertz time-domain spectroscopy (THz-TDS), optical-pump terahertz-probe (OPTP) photoconductivity spectroscopy, details on determination of background hole density, fitting methodology for IR reflectivity spectra, and parameters extracted from fits (PDF)
