# Supplementary material for: Optically Determined Hole Effective Mass in Tin-Iodide Perovskite Films
Source: ACS Energy Lett. 2025 Aug 28;10(9):4589–95. doi: 10.1021/acsenergylett.5c02283 (PMC12442082; doi:10.1021/acsenergylett.5c02283)
Supplement: Supplementary file 1 [file nz5c02283_si_001.pdf]

Supporting Information: Optically Determined Hole Effective Mass in Tin-Iodide Perovskite Films

Vincent J.-Y. Lim[1], Marcello Righetto[1], Michael D. Farrar[1], Thomas Siday[2], Henry J. Snaith[1], Michael B. Johnston[1], Laura M. Herz[1,*]

[1] Department of Physics, Clarendon Laboratory, University of Oxford, Parks Road, Oxford, OX1 3PU, U.K.

[2] School of Physics and Astronomy, University of Birmingham, Birmingham, B15 2TT, U.K.

Corresponding Author: laura.herz@physics.ox.ac.uk

1 Sample fabrication details

Sample preparation was analogous to that described recently in our other studies of tin iodide perovskites,[1,2] which also contain further characterization data including scanning electron microscopy images, UV-VIS absorption and x-ray diffraction and their changes following air exposure.

1.1 Solution preparation

Two 1.4 M stock solutions of $FASnI_3$ were made with 20% SnF_2 and 0% SnF_2. These were prepared 2 hours before the experiment, in the mixed solvent DMF:DMSO with a ratio of 4:1. For the 0% SnF_2 solution of $FASnI_3$, 963.03 mg of Formamidinium Iodide (Greatcell Solar), 2086.11 mg of Tin (II) Iodide (TCI) were dissolved in 4 mL. For the 20% SnF_2 solution of $FASnI_3$, 722.27 mg of FAI, 1564.58 mg of Tin (II) Iodide and 131.64 mg of Tin (II) fluoride (SnF_2, Sigma Aldrich) were dissolved in 3 mL.

The solutions were then mixed, and subsequent solution diluted, in the following manner to give the desired % Sn content:

**#**	**SnF_2 content (%)**			
1	0	-	-	
		**$FASnI_3$ (µL) 1% SnF_2**	**$FASnI_3$ (µL) 0% SnF_2**	**Total (µL)**
2	0.1	50	450	500
		**$FASnI_3$ (µL) 10% SnF_2**	**$FASnI_3$ (µL) 0% SnF_2**	**Total (µL)**
3	1	100	900	1000
		**$FASnI_3$ (µL) 20% SnF_2**	**$FASnI_3$ (µL) 0% SnF_2**	**Total (µL)**
4	10	1000	1000	2000
5	20	-	-	0

1.2 Sample preparation

Pre-cut quartz substrates were prepared by sonicating sequentially in Decon 90 detergent (1 % vol. in deionised (DI) water), DI water, Acetone and IPA for 10 minutes followed by UV Ozone treatment for 15 minutes.

Films were prepared on quartz using a single step anti-solvent quenching method in a nitrogen filled glovebox (H_2O <0.1 ppm, O_2 ≤ 1.0 ppm). 20 µL of the required solution was dispensed onto a static substrate and spun at 4000 rpm with an acceleration of 1000 rpm for a total of 40 seconds. At 20 s 100 µL of anisole was dispensed into the centre of the substrate at a height of roughly 5 mm from the surface in a steady stream taking around 1 second to fully dispense. Substrates were then transferred straight onto a hotplate and annealed for 10 minutes at 100 °C. All film preparation parameters were kept constant for each sample.

Samples for thickness determination via stylus profilometer (Dektak, Bruker) were spin coated onto glass substrates.

2 Experimental details and further characterisation data

2.1 Mid-IR reflectance and optical absorption

A Bruker 80v Fourier-transform infrared (FTIR) spectrometer was used for mid-IR reflectance and optical absorption measurements. For mid-IR reflectance, a nitrogen-cooled mercury cadmium telluride (MCT) detector, globar source, and KBr beamsplitter were used, and a gold mirror was used as a reference. For optical absorption, a silicon detector, tungsten lamp and CaF_2 beamsplitter were used. A silver mirror was used as the reference for reflection, and vacuum was used as the reference for transmission. A reflection-transmission geometry was used to obtain both reflectance and transmission, and the absorbance was calculated as

$$Absorbance = -\ln\left(\frac{T}{1-R}\right),$$

where T is the transmittance and R is the reflectance. We note that in this definition, *Absorbance* corresponds to the product αd of the absorption coefficient α of $FASnI_3$ and the film thickness *d,* i.e. it is defined by the natural logarithm of the light intensity ratios entering and leaving the thin film at its surfaces.

Figure S1 below shows the optical absorption spectra determined for the series of films with different SnF_2 concentration added during film fabrication. The absorption onset shifts to the blue for increasing hole doping (decreasing SnF_2 concentration) as previously observed,[1–3] owing to the Burstein-Moss effect.[4,5] Such effects result from significantly depletion of the available states near either the top of the valence band for p doping (holes), or the bottom of

the conduction band for n doping (electrons).[4,5] As a result, optical transitions are bleached near the band edge, leading to a blue shift of the onset of absorption, referred to as the Burstein-Moss shift.

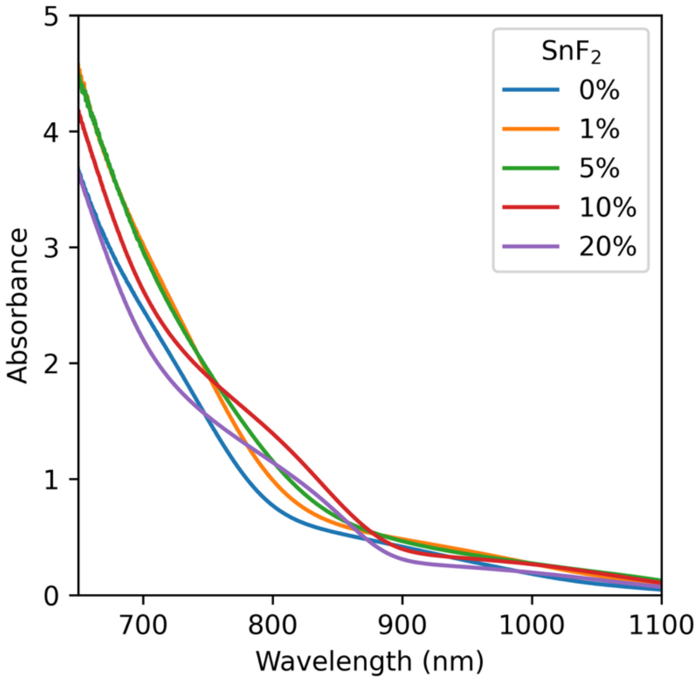

Figure S1 Absorbance spectra of $FASnI_3$ films with different concentration of SnF_2 added during film fabrication.

2.2 Terahertz time-domain spectroscopy (THz-TDS)

The experimental details for acquisition of THz-frequency IR absorption spectra have already been published in previous studies.[1,3,6] Briefly, an amplified Ti:Sapphire laser system was used for generation and detection of THz pulses, with characteristics ~ 35 fs pulse duration, 800 nm central wavelength, 5 kHz repetition rate (MaiTai – Ascend – Spitfire regenerative amplifier from Newport Spectra Physics). THz pulses were generated using a tri-layer spintronic emitter (2 nm tungsten, 1.8 nm $Co_{40}Fe_{40}B_{20}$, 2 nm platinum on a quartz substrate). These were focused onto the thin films deposited on z-cut quartz substrates using gold-coated off-axis parabolic mirrors, and then onto a 1 mm-thick (110)-ZnTe crystal for electro-optic sampling. The polarisation of the gate beam was detected with the combination of a quarter-wave plate, polarising beam splitter and a balanced photodiode. A home-made field-programmable gate

array-based board was used for data acquisition. Relative time delays were controlled using optical delay stages.

The measured time-domain transmission through the thin film was Fourier transformed into frequency space, $\tilde{T}_{sample}$, and compared to the transmission through the bare substrate $\tilde{T}_{substrate}$. The following formula was then used to calculate the dark conductivity spectrum of the thin film:[6–9]

$$\tilde{\sigma}_{\text{dark}} = \frac{\tilde{T}_{\text{substrate}} - \tilde{T}_{\text{sample}}}{\tilde{T}_{\text{sample}}} \frac{\epsilon_0 c(1+n_{\text{substrate}})}{d}$$

where $n_{\text{substrate}}$ is the refractive index of the substrate (2.1 in z-cut quartz substrates used for our study[10]), d is the thickness of the thin film sample, and c and ϵ_0 are the speed of light in free space and permittivity of free space, respectively. The thickness of the thin films was measured with a Dektak150 profiler. Only the low-frequency components (0.4 to 0.6 THz) of the spectra were used for determining p-doping densities in order to limit effects from optical phonon contributions.

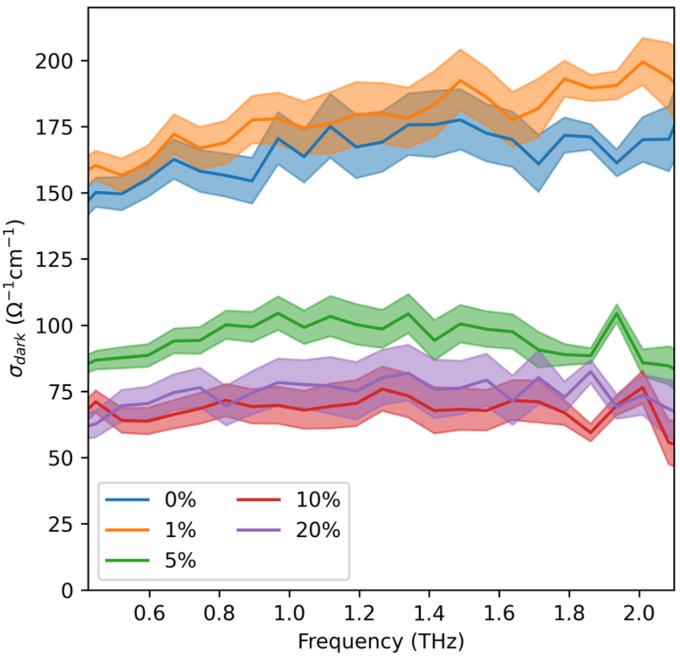

Figure S2 Dark conductivity spectra for $FASnI_3$ films with concentration of SnF_2 added during film fabrication.

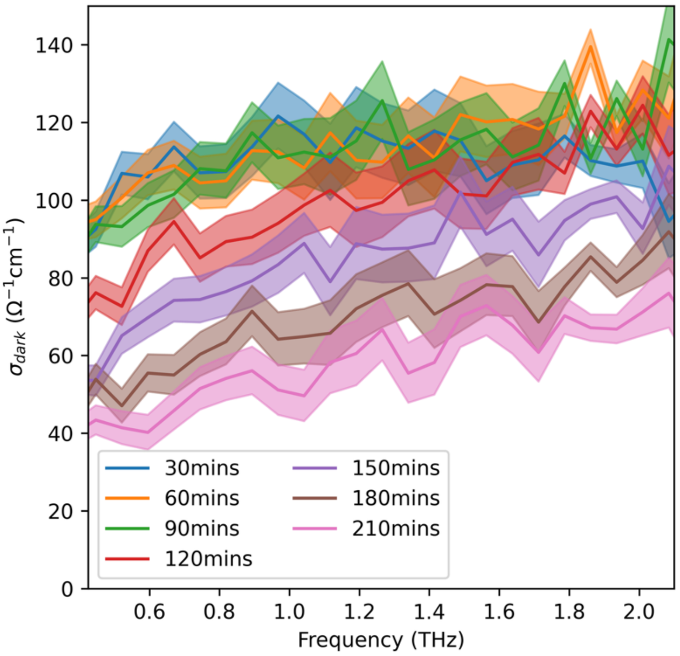

Figure S3 Dark conductivity spectra for an $FASnI_3$ film with 10% SnF_2 added during film fabrication, measured after a range of different time periods of exposure to ambient air. While the conductivity decreases over time in air, the mobility also decreases, as shown in Figure S5, with the overall effect being an increase in doping density, as shown in the main manuscript.

2.3 Optical-pump terahertz-probe (OPTP) conductivity spectroscopy

The same setup as for THz-TDS was used for OPTP, but now adding a 400nm pump pulse generated through frequency doubling of part of the fundamental laser output. The pump beam was expanded and was overlapping with the THz beam. The fluence was varied with a variable ND filter wheel.

The photoconductivity σ of the thin film sample is given by:[11]

$$\sigma = -\frac{\epsilon_0 c}{d}(1 + n_{substrate})\frac{\Delta T}{T} \ ,$$

where d is the thickness of the thin film, $n_{\mathrm{substrate}} = 2.1$ is the refractive index of the substrate at THz frequencies,[10] T is the transmitted THz radiation through the sample in the dark, ΔT is the difference in THz radiation between the photoexcited sample and the sample

under no illumination. ϵ_0 and *c* are free space permittivity and speed of light, respectively. The photoconductivity is also given by

$$\sigma(t) = n(t)e\mu$$

where $n(t)$ is the charge-carrier density at time t, e is the elementary charge, and μ is the electron-hole sum mobility. In this work, we assumed the mobilities of electrons and holes to be equal, as suggested by literature reporting theoretical values for bandstructure calculations and electron-phonon coupling.[12,13] While first-principles calculations may contain some errors in overall energy scaling, these are likely to apply relatively evenly across both bands, meaning that relative differentiation into electron and hole fractions are likely to be highly accurate. The charge-carrier density n at time = 0 can be derived from:

$$n(t = 0) = \frac{E\lambda}{hcA_{eff}d}$$

where A_{eff} is the effective area of the overlap between the THz probe and pump pulse, *E* is the energy of the photoexcitation pulse, λ is the wavelength of the photoexcitation (400 nm), and *h* is the Plank constant. Along with these equations and the maximum conductivity change when the pulse arrives (t=0) can be used to estimate the charge-carrier mobility, assuming a perfect absorption of the pump, and a full conversion of each photon to one electron-hole pair.

Changes in charge-carrier mobility values for different SnF_2 concentrations added during film fabrication, and following exposure to air, are shown in Figures S4 and S5, respectively. In each case, the mobilities decline with increasing hole concentration, most likely as a result of increasing hole interactions with other holes and ionized tin vacancies.

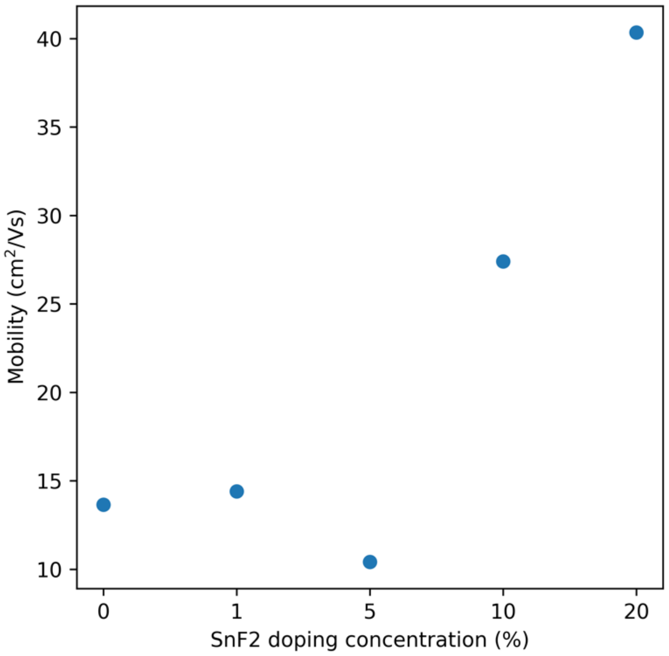

Figure S4 Charge-carrier mobility values extracted from OPTP measurements on $FASnI_3$ films with different SnF_2 concentrations added during film fabrication.

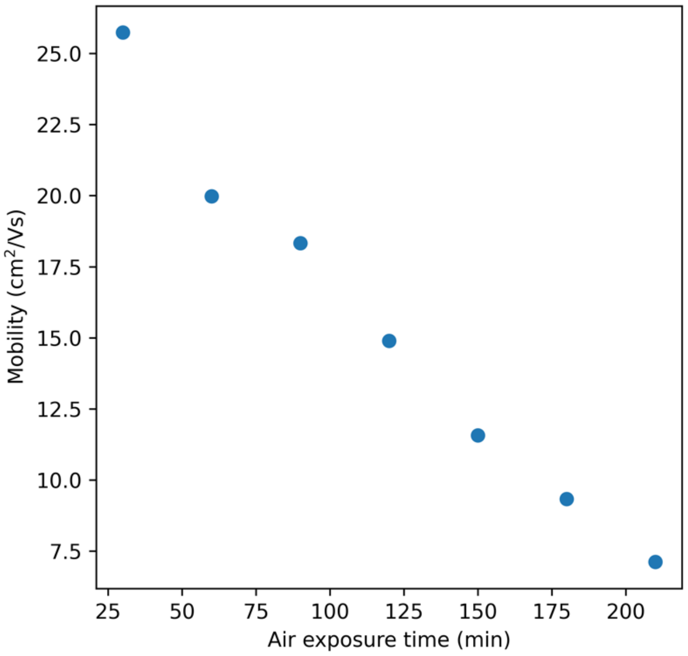

Figure S5 Charge-carrier mobility values extracted from OPTP measurements on $FASnI_3$ films (10% SnF_2 concentration) following different time periods of exposure to ambient air.

2.4 Determination of the background hole density

We convert the dark conductivity values σ_{dark} determined as described in Section 2.2. to background hole densities p in combination with measurements of the THz mobility described in Section S2.3. We utilise the reports of comparable electron and hole mobilities[12,13] to set the hole mobility μ_h as half the THz sum mobility value obtained OPTP (Figure S4), and then calculate the hole density p determined via the equation $\sigma_{dark} = pe\mu_h$. The resulting background hole densities range up to just above 10^{20}cm^{-3} as shown in the main manuscript.

3 Fitting and errors

The IR reflectivity spectra were modelled based on the Drude-Lorenz model assuming the presence of a plasma response (zero-frequency resonance) capturing the hole plasma, and a Lorentz oscillator term describing the internal vibration of the FA cation. In this model, the dielectric function of a doped semiconductor is as follows:[14,15]

$$\epsilon = \epsilon_{inf}\left(1 - \frac{\omega_p^2}{\omega^2 + i\omega\Gamma}\right) + \frac{A^2}{\omega_{FA}^2 - \omega^2 - i\omega\Gamma_{FA}}$$

where ω is the angular frequency (with $\omega = 2\pi\nu$ and ν being the frequency of the incident light), ϵ_{inf} is the dielectric constant at infinite angular frequency, ω_p is the plasma frequency, Γ and Γ_{FA} are the broadening factors for charge carriers and the FA vibrational mode respectively, A is the oscillator strength of the vibration, and ω_{FA} is the angular frequency of the FA internal vibrational mode. The plasma frequency is defined as $\omega_p^2 = pe^2/\epsilon_0\epsilon_{inf}m_{eff}$, where p is the doping (hole) density, ϵ_0 is the vacuum permittivity, and m_{eff} is the effective mass of the dopant charge carrier, i.e. the hole in this instance.

The reflectivity from the film at normal incidence was given by Fresnel reflection:

$$R = \left|\frac{1-n}{1+n}\right|^2,$$

where $n = \sqrt{\epsilon}$ is the refractive index function of the film.

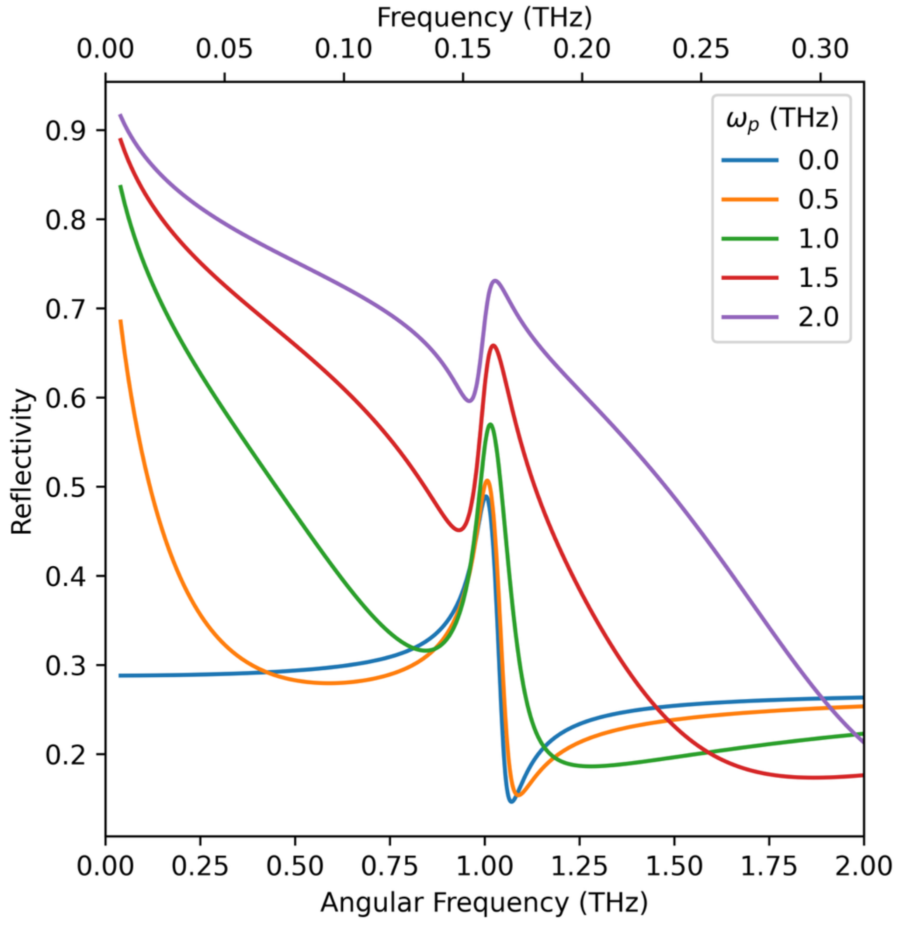

Figure S6 Simulated reflectivity spectra for a film with $A = 1$, ϵ_{inf} =10, $\Gamma = 1$radTHz, ω_{FA}=1 radTHz, Γ_{FA}=0.05 radTHz, for a range of different plasma frequencies indicated in the legend. Note that while the plasma frequency is as usual provide as an angular frequency ω (e.g. in Figure 3 of the main manuscript), the IR spectra (shown in Figure 2 of the main manuscript) are given in standard frequency $\nu = \omega/2\pi$.

Figure S6 shows simulated reflectivity spectra for films with parameters indicated in the caption, to demonstrate the expected change in the shape of the molecular vibrational mode as the plasma frequency is sweeping through the molecular resonance. Note that the top x-axis refers to standard frequency ν while the bottom axis indicates angular frequency $\omega = 2\pi\nu$. Conventionally, standard frequency is used to denote incident IR photon frequency (as in Figure 2 of the main manuscript) while the model for the dielectric function (and hence the plasma frequency ω_p) is given, as typical, in terms of angular frequency $\omega = 2\pi\nu$.

As described in the main text, all parameters were fitted globally across the full set of spectra, with the exceptions being a linear offset and the plasma frequency, which were allowed to

vary for each individual spectrum. The following are the parameters were extracted from global fits:

Fitting Parameter	Value
A	7.67×10^{13}
ϵ_{inf}	8.0 ± 1.0
Γ (rad s^{-1})	4.19×10^{14}
ω_{FA} (rad s^{-1})<br>ν_{FA} (THz)	3.22×10^{14}<br>51.2
Γ_{FA} (rad s^{-1})	1.66×10^{12}

Table S1 Table of extracted parameters globally varied across fits to all reflectivity spectra of $FASnI_3$ films.

We applied a linear offset to fits to the reflectivity spectra of each of the films, to incorporate effects arising from differences in reflection from the substrate and diffuse scattering from the film surface. This offset mostly affects determination accuracy of ϵ_{inf}, so we estimate the error in ϵ_{inf} by globally fitting the reflectivity spectra for the $FASnI_3$ film with 10% SnF_2 recorded across different air exposure times, while instead fixing the linear offset amount. Such fits resulted in $\epsilon_{inf} = 9.0$, which we take as the largest variation in ϵ_{inf} with respect to the value shown on Table S1, and therefore use to determine its error.

For the overall error estimation for the hole effective mass determination, the error from the linear fit, including the contributions of the error of each data point, and the uncertainty in the value of the ϵ_{inf} were added in quadrature. The errors associated with each data point include errors from mobility measurements from OPTP and conductivity measurements from THz-TDS, which were added in quadrature. Possible systematic errors could have arisen from the FASnI3 film with 20 mol% SnF_2 having non-zero doping density, although the satisfactory linear fit through the origin in Figure 3 of the main text suggests the doping density is indeed negligible. In addition, despite repeated reports of balanced electron and hole masses and mobilities in metal halide perovskites,[12,13] a small imbalance could add some error in the

separation of the overall hole mobility from the electron-hole sum mobility determined with OPTP.

References

(1) Lim, V. J. Y.; Ulatowski, A. M.; Kamaraki, C.; Klug, M. T.; Miranda Perez, L.; Johnston, M. B.; Herz, L. M. Air-Degradation Mechanisms in Mixed Lead-Tin Halide Perovskites for Solar Cells. *Adv. Energy Mater.* **2023**, *13* (33), 2200847.

(2) Savill, K. J.; Ulatowski, A. M.; Farrar, M. D.; Johnston, M. B.; Snaith, H. J.; Herz, L. M. Impact of Tin Fluoride Additive on the Properties of Mixed Tin-Lead Iodide Perovskite Semiconductors. *Adv. Funct. Mater.* **2020**, *30* (52), 2005594.

(3) Milot, R. L.; Klug, M. T.; Davies, C. L.; Wang, Z.; Kraus, H.; Snaith, H. J.; Johnston, M. B.; Herz, L. M.; Milot, R. L.; Klug, M. T.; Davies, C. L.; Wang, Z.; Snaith, H. J.; Johnston, M. B.; Herz, L. M.; Kraus, H. The Effects of Doping Density and Temperature on the Optoelectronic Properties of Formamidinium Tin Triiodide Thin Films. *Adv. Mater.* **2018**, *30* (44), 1804506.

(4) Burstein, E. Anomalous Optical Absorption Limit in InSb. *Phys. Rev.* **1954**, *93* (3), 632.

(5) Moss, T. S. The Interpretation of the Properties of Indium Antimonide. *Proc. Phys. Soc. Sect. B* **1954**, *67* (10), 775.

(6) Ulatowski, A. M.; Herz, L. M.; Johnston, M. B. Terahertz Conductivity Analysis for Highly Doped Thin-Film Semiconductors. *J. Infrared Millim. Terahertz Waves* **2020**, *41* (12), 1431–1449.

(7) Tinkham, M. Energy Gap Interpretation of Experiments on Infrared Transmission through Superconducting Films. *Phys. Rev.* **1956**, *104* (3), 845–846.

(8) Glover, R. E.; Tinkham, M. Conductivity of Superconducting Films for Photon Energies between 0.3 and 40kT_C. *Phys. Rev.* **1957**, *108* (2), 243–256.

(9) Ulatowski, A. M.; Farrar, M. D.; Snaith, H. J.; Johnston, M. B.; Herz, L. M. Revealing Ultrafast Charge-Carrier Thermalization in Tin-Iodide Perovskites through Novel Pump–Push–Probe Terahertz Spectroscopy. *ACS Photonics* **2021**, *8* (8), 2509–2518.

(10) Davies, C. L.; Patel, J. B.; Xia, C. Q.; Herz, L. M.; Johnston, M. B. Temperature-Dependent Refractive Index of Quartz at Terahertz Frequencies. *J. Infrared Millim. Terahertz Waves* **2018**, *39* (12), 1236–1248.

(11) Wehrenfennig, C.; Liu, M.; Snaith, H. J.; Johnston, M. B.; Herz, L. M. Charge-Carrier Dynamics in Vapour-Deposited Films of the Organolead Halide Perovskite $CH_3NH_3PbI_{3-x}Cl_X$. *Energy Environ. Sci.* **2014**, *7* (7), 2269–2275.

(12) Umari, P.; Mosconi, E.; De Angelis, F. Relativistic GW Calculations on $CH_3NH_3PbI_3$ and

$CH_3NH_3SnI_3$ Perovskites for Solar Cell Applications. *Sci. Rep.* **2014**, *4* (1), 4467.

(13) Poncé, S.; Schlipf, M.; Giustino, F. Origin of Low Carrier Mobilities in Halide Perovskites. *ACS Energy Lett.* **2019**, *4* (2), 456–463.

(14) McMahon, T. J.; Bell, R. J. Infrared Reflectivity of Doped InSb and CdS. *Phys. Rev.* **1969**, *182* (2), 526.

(15) Shkerdin, G.; Rabbaa, S.; Stiens, J.; Vounckx, R. Influence of Electron Scattering on Phonon–Plasmon Coupled Modes Dispersion and Free-Electron Absorption in n-Doped GaN Semiconductors at Mid-IR Wavelengths. *Phys. status solidi* **2014**, *251* (4), 882–891.
